# Global trends and inequalities in the burden of drug use disorders: a comprehensive analysis from 1990 to 2021 with future projections

**DOI:** 10.3389/fpubh.2025.1655575

**Published:** 2025-10-30

**Authors:** Jian Zhou, Menglin He, Guojun Zhou, Taoran Yang, Rurong Wang, Xuehan Li

**Affiliations:** ^1^Department of Anesthesiology, West China Hospital, Sichuan University, Chengdu, China; ^2^Department of Emergency, Binzhou Medical University Hospital, The First School Clinical Medicine of Binzhou Medical University, Binzhou, Shandong Province, China

**Keywords:** drug use disorders, global burden, opioid epidemic, health inequality, public health intervention

## Abstract

**Background:**

Drug use disorders (DUDs) pose a major global health challenge, with limited comprehensive data across demographic and socioeconomic groups.

**Methods:**

Using data from the Global Burden of Disease (GBD) 2021, we analyzed disability-adjusted life years (DALYs), deaths, prevalence, and incidence of DUDs from 1990 to 2021, stratified by sex, age, country, and Socio-Demographic Index (SDI). Trends were assessed using Joinpoint regression analysis, while cross-inequalities were evaluated through the Slope Index of Inequality (SII) and Concentration Index (CI). Nordpred projected future burden.

**Results:**

From 1990 to 2021, DALYs increased by 14.7%. Males and individuals aged 25–29 experienced the highest burden. High-SDI countries recorded the greatest DALYs and deaths. The SII increased from 82.4 in 1990 to 289.24 in 2021, and the CI revealed a disproportionate concentration of DUDs burden in high-SDI countries. Projections suggest that by 2044, DALYs will rise by 12.9 million, mainly due to opioid use disorders.

**Conclusion:**

The global burden of DUDs has increased significantly with widening health inequalities across SDI levels. Targeted interventions, particularly addressing the opioid crisis, are essential to manage and mitigate future impacts.

## 1 Introduction

Drug use disorders (DUDs) constitute a substantial global health concern, characterized by the non-medical misuse of psychoactive substances leading to dependence on drugs such as opioid, cocaine, amphetamine and cannabis (1). The widespread use of these substances has escalated dramatically in recent decades, with an estimated 250 million people worldwide using drugs in 2020, and approximately 36 million developing DUDs (2). Beyond their individual impact, DUDs contribute to broader public health crises by driving the spread of infectious diseases like HIV/AIDS and hepatitis, particularly among individuals who inject drugs and share contaminated paraphernalia (3).

The global burden of DUDs is profound, accounting for approximately 1.6% (1.3–2.1) of all-cause disability-adjusted life-years (DALYs) globally, reflecting a 21.8% increase from 1990 to 2019 (4). This burden varies significantly across populations, influenced by factors such as age, sex, genetic susceptibility, geographic location, socioeconomic conditions, and national drug policies. In 2013, China and India recorded DALYs of 182.9 and 119.6 per 100,000 people respectively, with only moderate changes from 1990 (5). In South America, where countries like Uruguay have legalized cannabis and Colombia, Peru, and Bolivia remain primary cocaine suppliers, the burden of DUDs has remained severe (6). However, national standardized treatment programs in these countries have improved the situation, underscoring the critical need for enhanced DUDs monitoring systems. In addition, the prevalence of DUDs has surged among younger populations in Europe, with a male-to-female ratio of 1.5 for years lived with disability (YLD) rates for DUDs (7). The patterns of DUDs are positively related to individual disability and mental diseases (8, 9). Across countries and regions, disparities in the burden of DUDs have widened, driven by the interplay of social, economic, and policy factors. Despite these alarming trends, comprehensive global data related to the burdens of DUDs from the global and overall point of view remain insufficient.

The Global Burden of Disease (GBD) database, managed by the Institute for Health Metrics and Evaluation (IHME), serves as a critical resource for governments, organizations, and researchers to prioritize health interventions and allocate resources effectively. International agencies such as the World Health Organization (WHO) and the World Bank also rely on GBD data to monitor progress toward health-related Sustainable Development Goals (SDGs) (10). While previous studies have documented the global burden of DUDs, critical remain. Moving beyond a descriptive update, this study targets critical blind spots in the existing literature: the evolving trends of specific drug subtypes, the scarcity of long-term burden projections, and a comprehensive accounting of cross-national inequalities. We therefore conducted an integrated analysis of the global burden of DUDs from 1990 to 2021, employing joinpoint regression, future predictions, and inequality metrics. This approach allows us to pinpoint critical trend shifts, forecast the evolving burden to 2044, and quantify the stark socioeconomic gradients, thereby offering a nuanced foundation for proactive and equitable public health action.

## 2 Methods

### 2.1 Data source

The Global Burden of Disease (GBD) 2021 database, maintained by the Institute for Health Metrics and Evaluation (IHME), provides comprehensive data on health loss for 369 diseases, injuries, and impairments, as well as 87 risk factors across 204 countries and territories (4). The dataset spans the years 1990 to 2021 and integrates information from diverse sources, including vital registration systems, population surveys, health records and epidemiological studies. In this study, the estimates and their 95% uncertainty interval (UI) for disability-adjusted life-years (DALYs), deaths, prevalence, and incidence of drug use disorders (DUDs) were downloaded from GBD 2021. Social Demographic Index (SDI), constructed from three key indicators, including income per capita, educational attainment and total fertility rate, was used to examine health inequalities, optimize resource allocation, and ultimately improve population health across diverse global contexts (11). The SDI ranges from 0 to 1, with higher value meaning a higher level of socioeconomic development.

### 2.2 Joinpoint regression analysis

Joinpoint regression analysis was applied to identify significant temporal trends in the Age Standardized Rates (ASRs) of DUDs from 1990 to 2021. This technique fits a series of joined straight lines, or segments, to the data, allowing for changes in the slope at specific points, called “joinpoints.” A log-linear model was selected, as the outcome variables (DALYs, deaths, prevalence, and incidence) are typically right-skewed and better conform to a Poisson-like distribution. The grid search method was used to identify the optimal number and location of joinpoints. The maximum number of joinpoints was set to 5, consistent with the software’s default recommendation. Model selection was performed using the Monte Carlo permutation test with 4,499 permutations and overall significance level of *α* = 0.05. To account for multiple testing across DUDs subtypes, the False Discovery Rate (FDR) correction was applied. The heteroscedastic error option was set to “Standard Error (Calculated)” to accommodate potential variability in the ASRs across years. For each segment between joinpoints, the Annual Percent Change (APC) and its 95% confidence interval (CI) were calculated. To summarize the overall trend across the entire study period, the Average Annual Percent Change (AAPC) was computed as a weighted average of the APCs (12). The values in both APC and AAPC greater than 0 indicate an upward, meaning the measured indicator is increasing over time on a yearly basis, while the values less than 0 indicate a downward trend, meaning the decreasing over time. The analysis was conducted using the Joinpoint Regression Program 4.9.1.0, developed by the National Cancer Institute (NCI).

### 2.3 Nordpred prediction of DUDs

The Nordpred statistical tool was employed to predict the rates of burden of diseases and the number of new cases. Data for four measures were categorized into 5-year age groups and 5-year calendar periods from 1990 to 2021, enabling a detailed analysis of trends across different age cohorts over time. ASRs were calculated using the world standard population to ensure comparability across populations and over time. Nordpred uses a log-linear age-period-cohort (APC) model, which considers the effects of age, period (calendar time), and cohort (birth cohort) on burden of disease, to extrapolate recent trends using a power-5 link function to moderate growth rates and mitigate the risk of overestimating future burden (13). In our study, we analyzed the global burden of DUDs for 5 years (1992–1996, 1997–2001, …, and 2017–2021) and the prediction was conducted in the 5 years (2022–2026, 2027–2031, …, 2042–2046).

### 2.4 Cross-country inequalities analysis

This analysis, aligned with the World Health Organization’s framework for health inequality monitoring, is used to measure, analyze, and address health inequalities, with a special focus on low- and middle-income countries (14). The Slope Index of Inequality (SII) is a measure of absolute inequality that captures the gradient in health outcomes across populations ranked from the most disadvantaged to the most advantaged (15). To calculate the SII, country-level data were ranked based on their SDI value from the GBD 2021 database. A weighted least squares regression was then performed, using the health outcome of interest as the dependent variable and the fractional rank as the independent variable (16). Population weights were applied to account for the size of country’s population. A higher value of SII indicates greater inequality, with a steeper gradient between the least and most advantaged countries. Concentration Index (CI) is a measure used to assess relative health inequality across different SDI countries, which are assigned a cumulative proportion of the global population, ranging from the poorest to the richest (17). The value of CI is computed as twice the area between the concentration curve and the line of equality (the 45 degrees line from the bottom left to the top right of the graph), ranging from −1 to 1, with a negative value indicates that countries with low SDI level are disproportionately affected by disease burden, while a positive value indicates that countries with high SDI level are disproportionately affected.

## 3 Results

### 3.1 Global burdens of DUDs at the sex, age level

**Figure 1:**
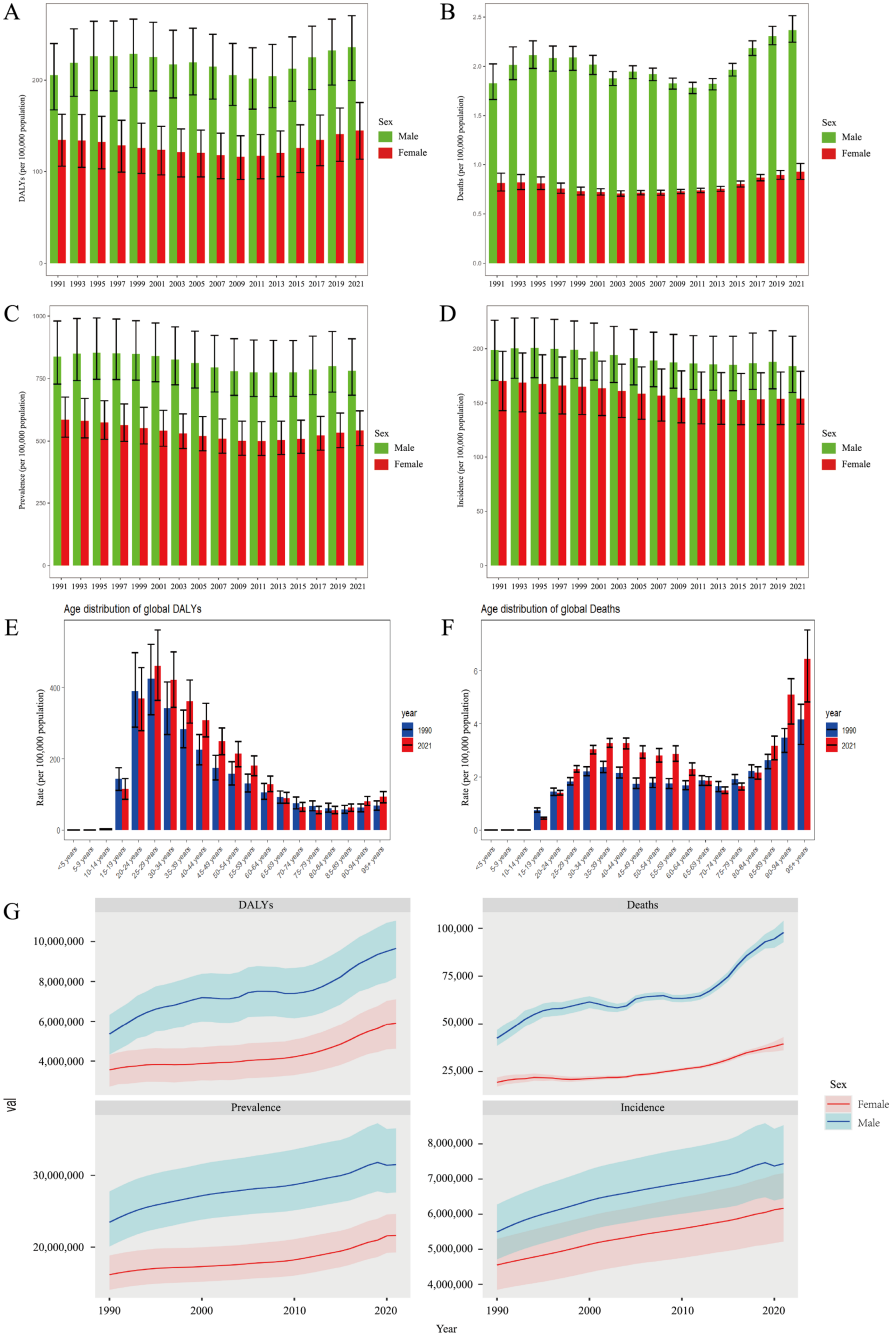
Global burden of DUDs per 100,000 population in **(A)** DALYs, **(B)** deaths, **(C)** prevalence, and **(D)** incidence by sex; **(E)** DALYs, and **(F)** deaths by age from 1990 to 2021; **(G)** total global burden for DUDs across the four measures. DUDs, drug use disorders; DALYs, disability adjusted life years.

The rate of DALYs attributed to DUDs increased notably between 1990 and 2021. For males, DALYs rose from 198.52 (UI: 161.30–234.34) to 235.88 (UI: 199.50–270.25), while for females, it increased from 133.73 (UI: 102.80–161.92) to 145.25 (UI: 113.42–175.63) (Figure 1A). A similar upward trend was observed in deaths related to DUDs, with male deaths rising from 1.74 (UI: 1.58–1.92) to 2.37 (UI: 2.24–2.51), and female deaths increasing from 0.79 (UI: 0.70–0.89) to 0.93 (UI: 0.85–1.01) (Figure 1B). Despite these trends in DALYs and deaths, no significant increase was observed in the rates of prevalence and incidence, although males continue to shoulder a disproportionately higher burden of DUDs compared to female (Figures 1C,D). When analyzing age-specific distributions, individuals aged 25–29 years consistently bore the highest DALYs in both 1990 and 2021 (1990: 424.88, UI: 324.73–521.56; 2021: 460.35, UI: 363.92–561.78) (Figure 1E). This was followed by those aged 20–24 and 30–34 years, indicating that the burden of DUDs is most prevalent among younger adults. As for deaths, the highest rates were observed in individuals aged 95 and older (1990: 4.16, UI: 3.22–4.73; 2021: 6.43, UI: 4.82–7.53), although younger age groups also exhibited a significant burden (Figure 1F). Overall, from 1990 to 2021, the number of DALYs, deaths, prevalence, and incidence attributed to DUDs has risen (Figure 1G). However, the male population consistently showed higher rates compared to females across all four measures.

### 3.2 Global burdens of DUDs at the national and SDI level

**Figure 2:**
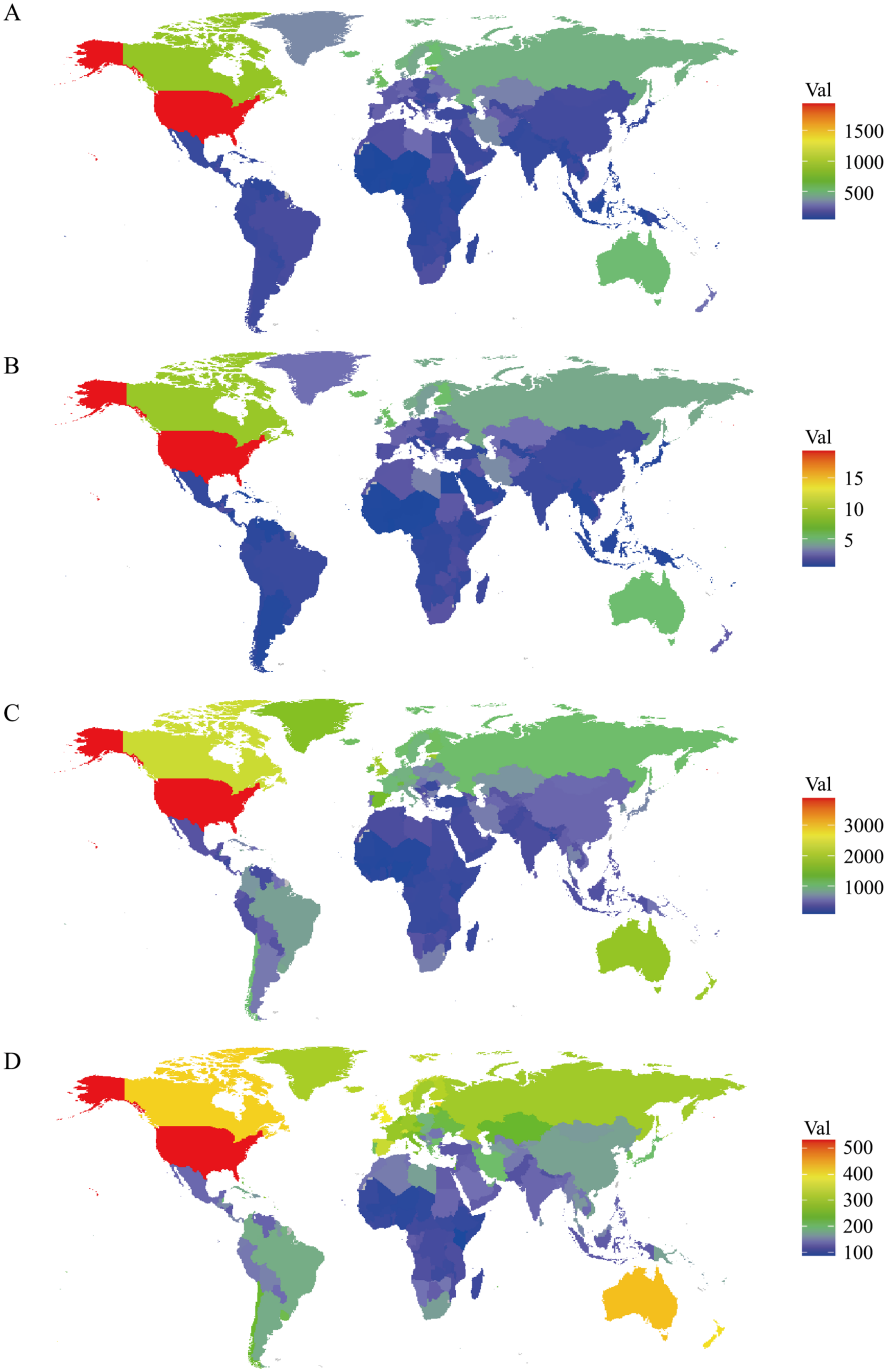
Age-standardized rates per 100,000 population for **(A)** DALYs, **(B)** deaths, **(C)** prevalence, and **(D)** incidence in 204 countries and territories.

In 2021, the United States (1944.08, UI: 1632.99, 2249.41), Canada (877.19, UI: 759.19, 998.21), and Estonia (733.92, UI: 586.79, 889.92) had the highest rates of DALYs attributed to DUDs (Figure 2A). Conversely, the countries with the lowest DALYs rates were Mali (36.87, UI: 25.47, 48.51), Guinea (35.54, UI: 25.19, 46.40) and Nigeria (33.93, UI: 24.41, 43.68) (Figure 2A). For deaths rates, Palestine (0.03, UI: 0.02, 0.03), Ghana (0.02, UI: 0.01, 0.03) and Palau (0.01, UI: 0.01, 0.01) recorded the lowest figures, while the United States (19.52, UI: 17.73, 21.61), Canada (8.85, UI: 8.08, 9.60), and Iceland (5.26, UI: 4.59, 5.93) were the top (Figure 2B). Additionally, the United States, Canada and United Kingdom were the top three countries with the highest prevalence rates of DUDs, and the United States, Australia, and Canada recorded the highest incidence rates of DUDs (Figures 2C,D). At the opposite end, Burkina Faso (228.58, UI: 193.64, 278.67), Nigeria (224.12, UI: 190.37, 260.09) and Togo (209.86, UI: 180.47, 248.47) had the lowest prevalence rates, while Togo (91.32, UI: 72.08, 107.44), Niger (90.93, UI: 75.51, 108.49) and Kenya (86.25, UI: 71.30, 101.66) showed the lowest incidence rates. In all analyses of DUDs burden, regions with higher SDI levels had the highest rates, while regions with lower SDI levels consistently had the lowest rates (Table 1).

**Table 1:** DALYs, deaths, prevalence, and incidence of drug use disorders by regions in 1990 and 2021, and percentage change over time.

Location	Male		Percentage change (%)	Female		Percentage change (%)
	Age-standardized rate (per 100,000 population)			Age-standardized rate (per 100,000 population)		
	1990	2021		1990	2021	
DALYs						
Low SDI	70.33 (55.16–88.83)	80.51 (63.63–97.85)	14.47%	52.28 (39.77–65.98)	52.96 (39.99–66.51)	1.30%
Low-middle SDI	81.98 (65.70–99.95)	94.24 (76.32–112.00)	14.95%	67.52 (51.95–84.67)	68.42 (51.20–85.78)	1.33%
Mid SDI	220.94 (182.11–258.23)	142.88 (115.77–169.35)	−35.33%	160.81 (125.04–195.33)	82.34 (61.86–102.13)	−48.80%
High-mid SDI	258.79 (209.09–303.51)	198.26 (162.11–233.43)	−20.26%	163.04 (124.63–197.37)	106.62 (79.90–132.82)	−34.61%
High SDI	278.92 (221.97–332.74)	910.89 (779.44–1048.47)	226.58%	163.80 (123.71–203.94)	584.29 (472.31.-696.97)	256.71%
Deaths						
Low SDI	0.67 (0.48–0.94)	0.81 (0.59–1.01)	20.90%	0.25 (0.21–0.29)	0.23 (0.20–0.26)	−8.00%
Low-middle SDI	0.65 (0.52–0.79)	0.86 (0.73–0.97)	32.31%	0.35 (0.30–0.39)	0.34 (0.30–0.39)	−2.86%
Mid SDI	2.43 (2.08–1,074)	1.21 (1.06–1.37)	−50.21%	1.21 (1.00–1.45)	0.35 (0.31–0.39)	−71.07%
High-mid SDI	2.22 (2.02–2.42)	1.52 (1.40–1.64)	−31.53%	0.92 (0.80–1.05)	0.39 (0.36–0.43)	−57.61%
High SDI	1.77 (1.70–1.84)	9.63 (8.96–10.50)	444.07%	0.66 (0.64–0.68)	4.39 (3.94–4.87)	565.15%
Prevalence						
Low SDI	416.55 (335.55–530.60)	413.58 (335.53–521.80)	0.71%	257.88 (218.46–313.00)	259.68 (220.04–313.75)	0.70%
Low-middle SDI	474.96 (393.30–590.95)	465.57 (393.63–569.27)	−1.98%	321.21 (275.50–382.76)	325.77 (280.46–386.89)	1.42%
Mid SDI	758.44 (650.08–894.17)	668.10 (569.62–794.71)	−11.91%	593.90 (519.09–685.95)	432.88 (373.07–508.58)	−27.11%
High-mid SDI	906.82 (785.89–1047.26)	795.19 (697.03–921.61)	−12.31%	649.05 (566.87–743.53)	528.71 (460.31–610.15)	−18.54%
High SDI	1544.66 (1339.04–1813.52)	2140.20 (1933.12–2417.72)	38.55%	1011.40 (872.82–1175.38)	1637.70 (1478.83–1826.72)	61.92%
Incidence						
Low SDI	120.66 (100.44–142.27)	126.75 (106.83–147.61)	5.05%	94.12 (77.68–112.25)	95.15 (79.47–112.25)	1.09%
Low-middle SDI	135.73 (115.01–158.42)	142.86 (121.69–164.41)	5.25%	113.51 (95.38–133.31)	118.04 (99.25–138.40)	3.99%
Mid SDI	189.07 (162.89–214.43)	170.16 (144.94–194.86)	−10.00%	167.51 (140.82–193.25)	139.36 (117.72–163.44)	16.80%
High-mid SDI	227.94 (197.83–259.18)	203.89 (176.41–231.37)	−10.55%	197.74 (164.68–232.15)	173.70 (144.93–203.13)	−12.16%
High SDI	308.17 (264.03–357.21)	374.78 (329.24–426.36)	21.61%	259.64 (218.46–304.40)	325.16 (282.60–372.61)	25.23%

### 3.3 Trends in global burdens of DUDs

**Figure 3:**
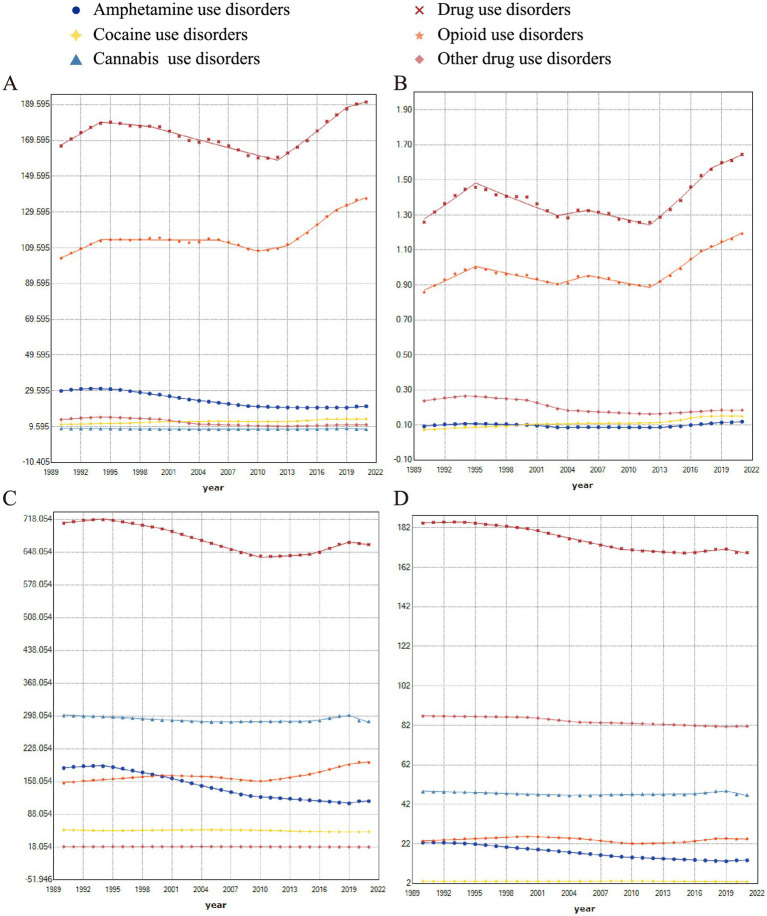
Joinpoint regression analysis for **(A)** DALYs, **(B)** deaths, **(C)** prevalence, and **(D)** incidence of DUDs, broken down by five subgroups: amphetamine, cannabis, cocaine, opioid, and other drugs, at the global level.

The study evaluated the change in rates of global burdens of DUDs from 1990 to 2021, and further included subgroup analysis of amphetamine, cannabis, cocaine, opioid, and other drug use disorders to estimate their contributions to overall DUDs burden. The analysis revealed an increasing trend in DALYs for cocaine use disorders (AAPC, 0.8), DUDs (AAPC, 0.4), and opioid use disorders (AAPC, 0.9) (Figure 3A). In contrast, amphetamine use disorders (AAPC, −1.1) and other drug use disorders (AAPC, −0.8) showed a declining trend. Similar patterns were observed in the analysis of deaths rates (Figure 3B). In terms of prevalence, amphetamine use disorders (AAPC, −1.5), cannabis use disorders (AAPC, −0.2), cocaine use disorders (AAPC, −0.2), DUDs (AAPC, −0.2), and other drug use disorders (AAPC, −0.1) exhibited a decreasing trend, while opioid use disorders (AAPC, 0.8) showed the faster growth (Figure 3C). Furthermore, the incidence of opioid use disorders (AAPC, 0.1) also increased, while the incidence of amphetamine use disorders (AAPC, −1.6), cannabis use disorders (AAPC, −0.2), cocaine use disorders (AAPC, −0.2), DUDs (AAPC, −0.3) and other drug use disorders (AAPC, −0.2) demonstrated a declining trend (Figure 3D). The detail of optimal joinpoints and corresponding APCs were shown in Table 2.

**Table 2:** Global APCs in ASR of DUDs and subgroups.

Measure	Drug use disorders		Amphetamine use disorders		Cannabis use disorders		Cocaine use disorders		Opioid use disorders		Other drug use disorders	
	Segment	APC	Segment	APC	Segment	APC	Segment	APC	Segment	APC	Segment	APC
DALYs	1990–1994	1.9* (1.2, 2.5)	1990–1993	1.4* (0.5, 2.2)	1990–2004	−0.3* (−0.4, −0.3)	1990–1997	0.9* (0.6, 1.2)	1990–1994	2.4* (1.8, 3)	1990–1994	2.4* (1.4, 3.4)
	1994–1999	−0.3 (0.9, 0.4)	1993–1996	−0.6 (−2.2, 1)	2004–2015	0 (0, 0.1)	1997–2000	2.1* (0, 4.3)	1994–2006	0 (−0.1, 0.1)	1994–2000	−1.4* (−1.9, −0.9)
	1999–2012	−0.9* (−1, 0.7)	1996–2009	−2.8* (−2.9, −2.7)	2015–2019	1.1* (0.8, 1.4)	2000–2006	0.4 (0, 0.9)	2006–2010	−1.4* (−2.3–0.5)	2000–2004	−5.5* (−6.3, −4.8)
	2012–2019	2.5* (2.1, 2.8)	2009–2013	−1.0* (−1.8, −0.2)	2019–2021	−2.6* (−3.2, −2)	2006–2013	−0.3* (−0.6, 0)	2010–2013	0.9 (−0.8, 2.8)	2004–2013	−1.3* (−1.5, −1.2)
	2019–2021	0.8(−1.1, 2.8)	2013–2019	0 (−0.3, 0.4)			2013–2017	2.9* (2, 3.8)	2013–2018	3.4* (2.9, 4)	2013–2018	1.7* (1.3, 2.1)
			2019–2021	1.9* (0.4, 3.4)			2017–2021	0.1 (−0.4, 0.6)	2018–2021	1.7* (0.8, 2.6)	2018–2021	0.2 (−0.5, 0.8)
Deaths	1990–1995	3.0* (2, 4.1)	1990–1994	3.6* (1.4, 5.9)	\		1990–2001	3.3* (2.7, 4)	1990–1995	2.9* (2, 3.9)	1990–1994	2.8* (1, 4.8)
	1995–2003	−1.7* (−2.1, −1.3)	1994–2000	−1.3* (−2.3, −0.2)	\		2001–2013	0.6* (0.2, 0.9)	1995–2003	−1.3* (−1.6, −1)	1994–2000	−1.7* (−2.5, −0.8)
	2003–2006	0.7 (−1.2, 2.7)	2000–2003	−4.7* (−8.4, −1)	\		2013–2017	7.2* (5.4, 9.1)	2003–2006	1.8* (0.3, 3.3)	2000–2004	−6.8* (−7.9, −5.6)
	2006–2012	−1.1* (−1.5, −0.7)	2003–2013	−0.1 (−0.3, 0.1)	\		2017–2021	0.6 (−0.9, 2.1)	2006–2012	−1.3* (−1.6, −0.9)	2004–2012	−1.5* (−1.7, −1.4)
	2012–2018	4.0* (3.5, 4.4)	2013–2019	4.7* (4.2, 5.3)	\				2012–2017	4.2* (3.7, 4.8)	2012–2019	1.9* (1.7, 2.1)
	2018–2021	1.6 (−0.1, 3.3)	2019–2021	1.8 (−2.3, 6)	\				2017–2021	2.4* (1.6, 3.2)	2019–2021	−0.1 (−1.6, 1.3)
Prevalence	1990–1994	0.3* (0.2, 0.4)	1990–1994	0.6* (0.4, 0.9)	1990–2005	−0.3* (−0.4, −0.3)	1990–1994	−0.8* (−1, −0.6)	1990–2000	0.9* (0.9, 1)	1990–2000	0.0* (0, 0)
	1994–2000	−0.5* (−0.5, −0.4)	1994–2000	−2.1* (−2.2, −1.9)	2005–2015	0.1 (0, 0.1)	1994–2005	0.3* (0.3, 0.4)	2000–2005	−0.4* (−0.6, −0.1)	2000–2004	0.1* (0.1, 0.2)
	2000–2010	−0.9* (−0.9, −0.9)	2000–2009	−3.2* (−3.3, −3.1)	2015–2019	1.1* (0.8, 1.4)	2005–2011	−0.5* (−0.6, −0.4)	2005–2010	−1.3* (−1.5, −1)	2004–2008	−0.2* (−0.2, −0.1)
	2010–2015	0.2* (0.1, 0.3)	2009–2019	−1.2* (−1.3, −1.1)	2019–2021	−2.5* (−3.2, −1.9)	2011–2015	−1.1* (−1.3, −0.9)	2010–2015	1.9* (1.6, 2.1)	2008–2014	−0.3* (−0.4, −0.3)
	2015–2019	1.0* (0.9, 1.1)	2019–2021	2.0* (1.2, 2.8)			2015–2019	−0.5* (−0.7, −0.3)	2015–2019	3.0* (2.7, 3.4)	2014–2019	−0.2* (−0.2, −0.2)
	2019–2021	−0.4* (−0.6, −0.2)					2019–2021	0.5 (0, 1)	2019–2021	1.2* (0.4, 1.9)	2019–2021	0.1* (0, 0.2)
Incidence	1990–1994	0.1 (0, 0.1)	1990–1994	−0.5* (−0.7, −0.3)	1990–2004	−0.3* (−0.4, −0.3)	1990–1999	0.3* (0.2, 0.4)	1990–2000	0.9* (0.8, 1)	1990–2000	−0.1* (−0.1, −0.1)
	1994–2000	−0.3* (−0.4, −0.2)	1994–2003	−2.2* (−2.3, −2.1)	2004–2016	0.1* (0.1, 0.2)	1999–2004	−0.3* (−0.7, 0.1)	2000–2005	−0.9* (−1.3, −0.5)	2000–2005	−0.6* (−0.6, −0.6)
	2000–2009	−0.7* (−0.7, −0.6)	2003–2009	−2.8* (−2.9, −2.6)	2016–2019	1.0* (0.1, 1.9)	2004–2010	0.6* (0.3, 0.9)	2005–2010	−2.3* (−2.7, −1.9)	2005–2010	−0.1* (−0.2, −0.1)
	2009–2015	−0.2* (−0.2, −0.1)	2009–2019	−1.5* (−1.6, −1.5)	2019–2021	−2.4* (−3.2, −1.5)	2010–2021	−1.1* (−1.2, −1)	2010–2015	0.7* (0.3, 1.1)	2010–2019	−0.2* (−0.2, −0.2)
	2015–2019	0.3* (0.2, 0.4)	2019–2021	2.0* (1.4, 2.7)					2015–2018	2.6* (1.3, 3.9)	2019–2021	0.1* (0, 0.3)
	2019–2021	−0.6* (−0.9, −0.3)							2018–2021	−0.3 (−0.9, 0.3)		

*p < 0.05.

### 3.4 Prediction of global burdens of DUDs

**Figure 4:**
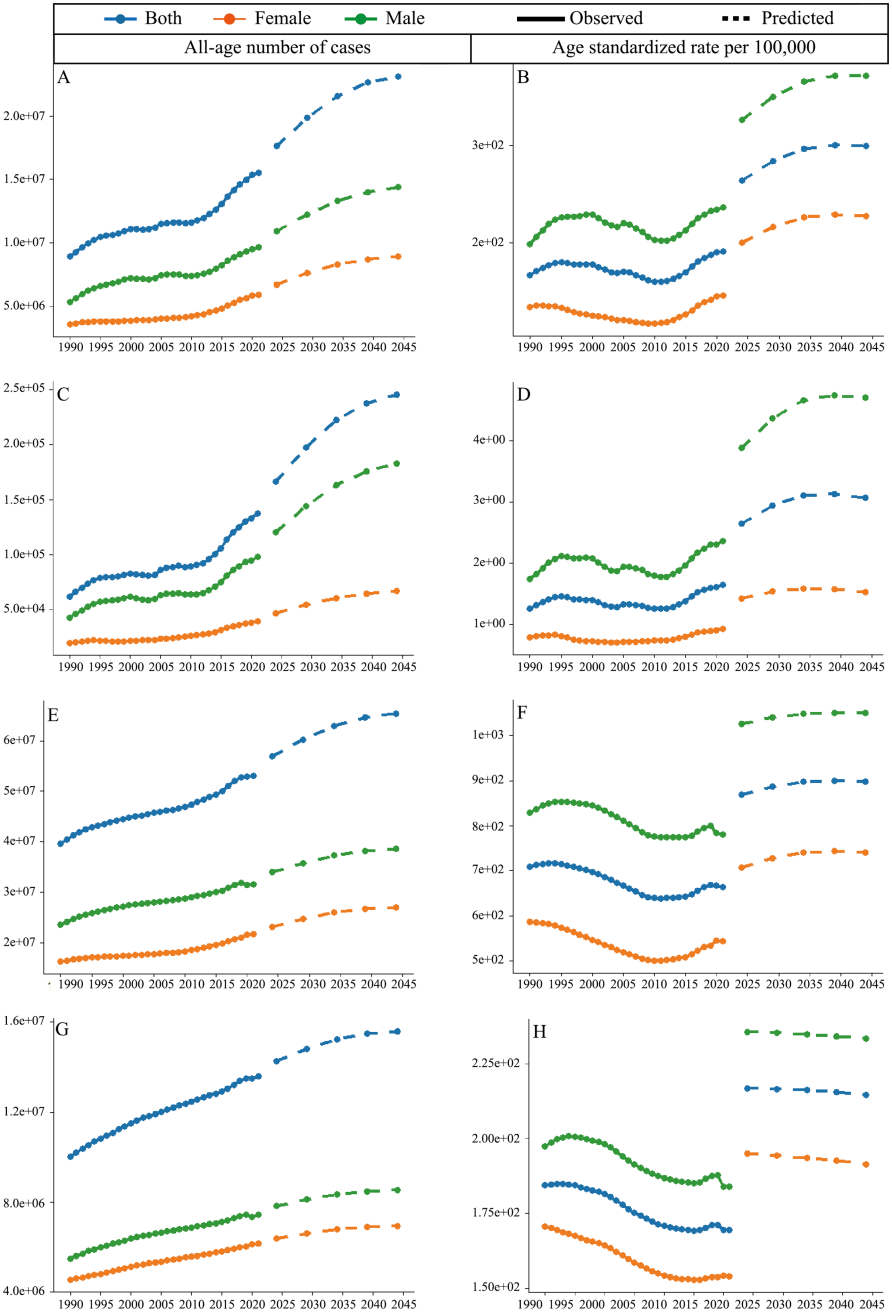
Predicted trends for DUDs. All-age number of cases for **(A)** DALYs, **(C)** deaths, **(E)** prevalence, and **(G)** incidence; age-standardized rate per 100,000 for **(B)** DALYs, **(D)** deaths, **(F)** prevalence, and **(H)** incidence.

The average annual all-age number of DALYs is projected to increase by 12.9 million from the 1992–1996 period to the 2042–2046 period (Figure 4A). The gap between males and females has also widened during this period. From 1990 to 2021, the age-standardized DALYs rate (ASDR) of DUDs showed an increasing but fluctuating trend; however, this increase is expected to accelerate from 2021 to 2044 (Figure 4B). A similar pattern was observed in the analysis of deaths, with an even wider gender gap (Figures 4C,D). As for prevalence, the average annual all-age number is expected to rise by 23 million from the 1992–1996 period to the 2042–2046 (Figure 4E). However, the age-standardized prevalence rate (ASPR) of DUDs decreased slowly from 1990 to 2021, which is predicted to grow from 2021 to 2044 (Figure 4F). Additionally, the increase in the average annual all-age number of incidence from the 2042–2046 period to the 2017–2021 period is nearly 0.76 times to that seen from the 2017–2021 period to the 1992–1996 period, indicating a slowly increase trend (Figure 4G). Of note is that age-standardized incidence rate (ASIR) of DUDs is predicted to decrease from the 2022–2026 period to the 2042–2046 period (Figure 4H).

### 3.5 Cross-country inequality analysis

**Figure 5:**
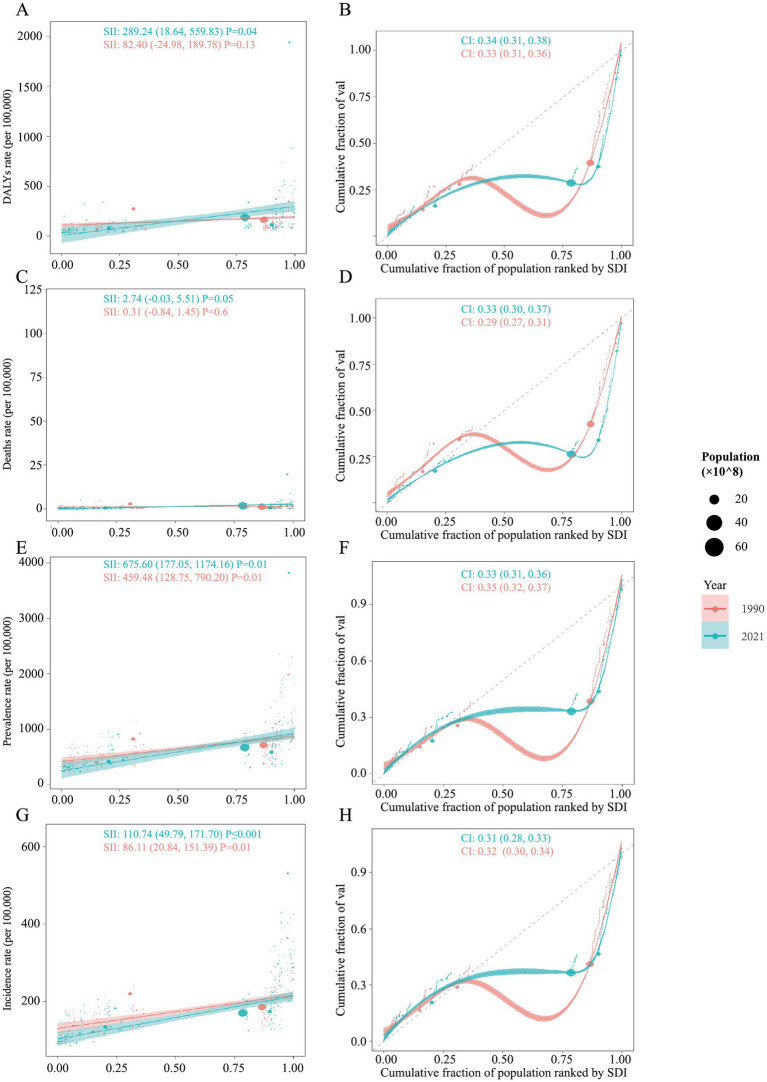
Health inequality analysis for the global burden of DUDs from 1990 to 2021. Regression curves (Slope Index of Inequality, SII) and concentration curves (Concentration Index, CI) are presented for **(A,B)** DALYs, **(C,D)** Deaths, **(E,F)** prevalence, and **(G,H)** incidence.

The analysis revealed notable changes in both absolute and relative health inequalities across the years, as indicated by shifts in the SII and CI values. In 1990, the distribution of DALYs across SDI rankings were more concentrated in countries with higher SDI (Figure 5A). The value of SII was 82.40, which means that there was an estimated burden of 82.40 (per 100,000 population) DALYs between country with the highest SDI and the lowest SDI in 1990, and this gap further amplified to 289.24 in 2021. Moreover, the value of CI also increased from 1990 to 2021, which means the relative health equalities are more disproportionately concentrated in countries with higher SDI (Figure 5B). However, no significant trend was observed in the analysis of deaths burden (Figures 5C,D). In the analyses of prevalence and incidence, despite the burdens were likely centered on countries with higher SDI, this increased from 459.48 in 1990 to 675.60 in 2021 for prevalence, and from 86.11 in 1990 to 110.74 in 2021 for incidence (Figures 5E,G). Meanwhile, no significant changes in relative SDI-related health inequalities were found for prevalence and incidence (Figures 5F,H).

## 4 Discussion

To the best of our knowledge, the findings of this study firstly demonstrate the significant and growing burden of drug use disorders (DUDs) worldwide, with substantial variation across age, sex, national, and social demographic index (SDI) levels from 1990 to 2021, providing a basis for targeted interventions. Notably, our analysis confirmed that male bear a heavier burden of DUDs than female across all measures, a trend that has persisted over time. While this may be partly explained by social environment, culture, or individual behavior, it also points to potential gaps in gender-sensitive approaches to prevention and treatment. The burden of DUDs is most concentrated among younger adults, especially those aged 25–29 years, for DALYs, which should be taken seriously, as this period is of great importance to their cognitive and emotional development. The study also reveals that high-SDI countries, such as the United States, Canada, and Estonia, shoulder the greatest DUDs burden, with significantly higher DALYs and deaths rates compared to lower-SDI countries. Moreover, different types of DUDs exhibited distinct trends: opioid and cocaine use disorders showed an increase in both DALYs and deaths, while amphetamine and other drug use disorders experienced a decline. From the 1992–1996 period to the 2042–2046 period, the average annual of global DALYs attributed to DUDs are projected to increase by 12.9 million It should be noted that the temporal trends show a widening gender gap, highlighting the need for gender-specific interventions.

Across all years studied, the burden of DUDs was consistently higher in males than in females. This disparity was reflected in DALYs, which were 1.48 and 1.62 times higher in males in 1990 and 2021, respectively, and was even more pronounced in mortality, with male death rates 2.22 and 2.55 times greater in 1990 and 2021. These differences might partly due to drug accessibility, as males have greater opportunities to use drug than female, especially in North American and Latin American (18, 19). However, DUDs among males are growing rapidly, and this available data might be underestimated for different reasons, such as cultural and religious stigma for reporting use of drug in males and so on (20). The accelerating disparity suggests that existing public interventions may be failing to engage effectively with male populations, which underscores an urgent need for gender-transformative interventions that move beyond one-size-fits-all approaches, specifically targeting masculine norms and barriers to healthcare access for men. As for analysis of age distribution, people aged from 25 to 29 shouldered heaviest burden of DUDs in both 1990 and 2021 for DALYs, followed by groups of 20–24 and 30–34 years, showing the character of this addiction affecting younger populations. This phenomenon is in line with previous GBD studies exploring substance use disorders among young people in Europe (21). DUDs at these ages can have far-reaching consequences, including long-term disability, social exclusion, and economic instability (22). In addition, a pivotal finding of our analysis if the pronounced and growing concentration of DUDs burden in high-SDI countries, as evidenced by substantial increase in both the SII and CI. This challenges the simplistic narrative that DUDs are primarily a problem of poverty. Instead, it points to a ‘high-income epidemic’ driven by distinct mechanisms. High-SDI countries, including the United States, Canada, and Australia, exhibit the highest rates of DUDs-related DALYs and deaths. This trend likely reflects the influence of healthcare systems, drug policies, and socio-economic factors. For example, the opioid crisis in the United States has been driven by systemic healthcare failures, including the over-prescription of pain medications and inadequate mental health support services (23). Of note, the illicit drug injection always intertwined with the HIV and hepatitis C virus epidemics, as a result of sharing of drug paraphernalia, impaired judgment, risk behaviors, weakened immune system and co-infection risk (24). And the burden of DUDs remains leading cause of DALYs in Australia both in 1990 and 2015, even with Australia government’s increased policy attention and investment (25). In contrast, while low-SDI countries experiencing lower overall rates of DALYs related to DUDs, some situations should be pointed out. In many low-SDI countries, the availability of illicit drugs may be lower due to less widespread distribution networks or stricter enforcement measures, which naturally reduces the prevalence of drug use disorders in these regions (26). In addition, healthcare infrastructure and data collection systems may be underdeveloped in low-SDI countries, leading to underreporting of drug use, as well as health systems often prioritize infectious diseases and maternal-child health over chronic conditions (27). This divergence underscores that the drivers and manifestations of DUDs are context-specific, demanding fundamentally different policy responses: addressing systemic healthcare and market failures in high-income nations, while strengthening foundational health information systems and integrated care in low-income settings. Then, we further revealed divergent trends in the burden of different types of DUDs, including Amphetamine, cannabis, cocaine, opioid and other drug use disorders to estimate their proportions contribute to DUDs. Opioid use disorder exhibited increasing trends in DALYs, deaths, prevalence, and incidence, establishing it as the principal driver of the rising global burden of DUDs and underscoring the critical urgency of the ongoing opioid epidemic. This might attribute to over-prescription of opioid (such as oxycodone and fentanyl) in North America, leading to a large number of people becoming addicted, and this problem is exacerbated by many patients switching from prescription drugs to illegal opioids even under stricter drug management policies in many countries (28). Our findings on the burden of opioid use disorders underscore the critical role of pharmacovigilance. Strengthening systems for post-market surveillance, spontaneous reporting, and signal detection detection-aligned with international guidelines-is essential. Integrating this regulatory data with public health metrics can provide an early-warning system to enable proactive interventions against prescription drug misuse. The decreasing trend of amphetamine use disorder might attribute to the restricted illicit production and circulation of amphetamines through strict laws and policies, the increasing usage of its substitute goods (opioids), and greater public awareness of the dangers of this drug (29). The trend in global burdens of cocaine use disorder varies among countries and regions. In south America, which is a major cocaine supplier, the burden of cocaine use disorder is still at a high level as a result of its particular profile of drug use (30). However, under the long-term national treatment programs over the past decade, the major cocaine production and distribution networks has been cracked down by international law enforcement cooperation, which limits the global supply of cocaine, leading to a decline in use in some areas (31). The legalization of cannabis, normally accompanied by a decline in awareness of its potential harms, has led to its use becoming more widespread, particularly among young people (32). Our projections indicate that the global burden of DUDs will continue to increase in the coming decades, with a significant rise in both DALYs and prevalence expected by 2044. This upward trend is particularly concerning given that existing public health efforts have been insufficient in curbing the rise of DUDs. Without substantial policy reforms, including expanded access to addiction treatment and prevention programs, the burden of DUDs is likely to place even greater strain on healthcare systems worldwide. Attention should also be paid to the fact that DUDs are often intricately correlated with alcohol use disorder, mental disorder and select disabilities, reflecting the complex interplay of these conditions (7, 9, 33). In synthesis, our findings reveal a triple crisis demanding differentiated responses. The high-income epidemic, evidenced by rising inequality indices, necessitates systemic reforms in high-SDI nations-addressing healthcare failures behind the opioid crisis and strengthening pharmaceutical regulation. Concurrently, the potential crisis in lower-SDI regions, masked by surveillance gaps, calls for preemptive investment in health infrastructure and integrated care to build resilience. Furthermore, persisting demographic disparities, notably youth and between genders, require life-course and gender-sensitive interventions. Confronting the multifaceted drivers of DUDs therefore mandates an integrated, cross-sectoral strategy that robustly combines prevention, treatment, and harm reduction.

### 4.1 Strengths and limitations

One of the primary strengths of this study lies in its utilization of the Global Burden of Disease (GBD) database, which offers comprehensive, standardized data on the burden of DUDs across 204 countries over a nearly three-decade period. This provides a robust foundation for identifying trends at the multiple levels, including sex, age, and SDI, and allows for both global and regional comparisons. Another strength is the inclusion of cross-country inequality analyses, which reveal disparities in the distribution of DUDs-related burden and underscore the need for targeted policies in both high- and low-SDI regions. Finally, by incorporating multiple metrics-DALYs, deaths, prevalence, and incidence, the study provides a comprehensive assessment of the multifaceted impact of DUDs on global health. However, several limitations must be acknowledged. First, despite the comprehensiveness of the GBD database, data quality and availability vary significantly across countries, particularly in low-SDI countries. This may result in underestimation or inaccurate reporting of DUDs-related burdens, as many countries lack robust healthcare infrastructure and data collection systems. Second, although we employed standard measure of inequality (SII and CI), we did not perform sensitivity analyses, such as using alternative SDI vintages or conducting regional sub-group rankings. Third, the future projections from the Nordpred model, while useful for indicating trends, are presented without prediction intervals and were not internally validated through back-testing. Finally, the study focuses primarily on aggregate, population-level data, which may obscure important within-country disparities, such as those related to socioeconomic status, urban–rural divides, and specific drug types. Further research incorporating individual-level data is needed to provide a more nuanced understanding of the factors driving DUDs and their health impacts.

## 5 Conclusion

The study highlights the complex and evolving nature of the global burden of drug use disorders (DUDs), with significant variations across sex, age, and socio-demographic factors. While high-income countries continue to bear the greatest burden, low-income nations are increasingly vulnerable to the long-term impacts of drug addiction. The rising burdens of opioid use disorders and the widening gender gap demand urgent action to develop comprehensive, evidence-based public health interventions. Addressing the global burden of DUDs will require a coordinated response, incorporating harm reduction, mental health support, and targeted policies to address the root causes of substance abuse across diverse populations.

## Data Availability

Publicly available datasets were analyzed in this study. This data can be found here: The data utilized in this study are publicly accessible through the official GBD 2019 website https://vizhub.healthdata.org/gbd-results/.

## Supplementary material

Supplementary Table The SDI values of locations from 1980 to 2021.
